# Spin-exchange carrier multiplication in manganese-doped colloidal quantum dots

**DOI:** 10.1038/s41563-023-01598-x

**Published:** 2023-07-13

**Authors:** Ho Jin, Clément Livache, Whi Dong Kim, Benjamin T. Diroll, Richard D. Schaller, Victor I. Klimov

**Affiliations:** 1 Nanotechnology and Advanced Spectroscopy Team, C-PCS, Chemistry Division, Los Alamos National Laboratory, Los Alamos, NM USA; 2 Center for High Technology Materials, University of New Mexico, Albuquerque, NM USA; 3 Center for Nanoscale Materials, Argonne National Laboratory, Lemont, IL USA; 4 Department of Chemistry, Northwestern University, Evanston, IL USA

**Keywords:** Quantum dots, Quantum dots, Optical spectroscopy

## Abstract

Carrier multiplication is a process whereby a kinetic energy of a carrier relaxes via generation of additional electron–hole pairs (excitons). This effect has been extensively studied in the context of advanced photoconversion as it could boost the yield of generated excitons. Carrier multiplication is driven by carrier–carrier interactions that lead to excitation of a valence-band electron to the conduction band. Normally, the rate of phonon-assisted relaxation exceeds that of Coulombic collisions, which limits the carrier multiplication yield. Here we show that this limitation can be overcome by exploiting not ‘direct’ but ‘spin-exchange’ Coulomb interactions in manganese-doped core/shell PbSe/CdSe quantum dots. In these structures, carrier multiplication occurs via two spin-exchange steps. First, an exciton generated in the CdSe shell is rapidly transferred to a Mn dopant. Then, the excited Mn ion undergoes spin-flip relaxation via a spin-conserving pathway, which creates two excitons in the PbSe core. Due to the extremely fast, subpicosecond timescales of spin-exchange interactions, the Mn-doped quantum dots exhibit an up-to-threefold enhancement of the multiexciton yield versus the undoped samples, which points towards the considerable potential of spin-exchange carrier multiplication in advanced photoconversion.

## Abstract (web summary)

Carrier multiplication generates multiple excitons for each absorbed photon but is normally limited by fast phonon-assisted relaxation. Here the authors achieve a threefold enhancement in multiexciton yields in Mn-doped PbSe/CdSe quantum dots, due to very fast spin-exchange interactions between Mn ions and the quantum dots that outpace energy losses arising from phonon emission.

## Main

During carrier multiplication (CM) a high-energy, ‘hot’ electron or hole relaxes within the same band by creating new electron–hole (e–h) pairs^1–6^. This occurs via one or more impact ionization events whereby a pre-existing valence-band electron is excited to the conduction band via an Auger-type collision with a high-energy carrier. In principle, CM can improve the performance of optoelectronic, photovoltaic (PV) and photocatalytic devices because due to this process, the quantum efficiency of the photon to e–h pair conversion (*Q*_eh_) becomes greater than one^7–13^. In the case of PVs, this would boost the photocurrent and, as a result, the power conversion efficiency could reach 44.4% instead of 33.7% in the case of *Q*_eh_ = 1 (refs. ^14–16^).

Due to restrictions imposed by energy and translational momentum conservation, in bulk semiconductors, the photon energy (*hv*; *h* is the Planck constant, and *v* is the photon frequency) required to trigger CM is at least four bandgaps (*E*_g_) and typically is much higher^9,17,18^. As a result, for most PV-relevant semiconductors, the onset of CM (*hv*_th_; *v*_th_ is the photon frequency at the CM threshold) is too high for this effect to provide a discernible contribution to a photocurrent. Due to the relaxation of the translational momentum conservation^19^, the CM threshold is reduced in zero-dimensional semiconductor quantum dots (QDs)^20–22^. In particular, in colloidal PbSe QDs, the CM onset is below 3*E*_g_ (ref. ^5^), versus >6.5*E*_g_ in bulk PbSe (refs. ^9,18^). A further decrease in *hv*_th_ has been obtained using specially engineered PbSe/CdSe (ref. ^23^) and PbS/CdS (ref. ^24^) hetero-QDs, for which the CM threshold drops to ~2*E*_g_—that is, it reaches the energy-conservation-defined limit.

Another important characteristic of CM is the e–h pair creation energy, *ε*_eh_ = (d*Q*_eh_/d*hv*)^−1^ (ref. ^25^). The inverse of *ε*_eh_ defines the steepness of the *Q*_eh_ growth, implying that for a fixed photon energy, smaller values of *ε*_eh_ lead to higher *Q*_eh_. Based on previous research, *ε*_eh_ is controlled by the interplay between the energy gain rate (*r*_gain,ii_) and energy loss rate (*r*_loss_) associated with, respectively, impact ionization and phonon emission^20^. In particular, near the CM threshold, *ε*_eh_ ≈ *E*_g_(*r*_loss_/*r*_gain,ii_), where *r*_gain,ii_ = *E*_g_/*τ*_ii_ and *r*_loss_ = *E*_phon_/*τ*_phon_; *τ*_ii_ is the characteristic time of a Coulombic collision (impact ionization), and *E*_phon_ and *τ*_phon_ are the characteristic phonon energy and the phonon emission time, respectively^20,26^. In bulk semiconductors, the minimal value of *ε*_eh_ is ~3*E*_g_ (ref. ^25^), suggesting that energy losses outpace energy gains by at least a factor of three. Together with the high CM threshold, this greatly limits the practical utility of the CM process.

Interestingly, while demonstrating a reduced CM threshold, QDs do not exhibit an appreciable reduction in *ε*_eh_ compared to bulk solids^3^. In particular, as was pointed out in the literature^26^, zero-dimensional confinement leads to a synchronous increase in the rates of both the Auger interactions underlying CM and the competing intra-band energy losses (likely also assisted by Auger-type processes^27,28^). As a result, the relationship between *r*_gain,ii_ and *r*_loss_ is not appreciably modified by quantum confinement and still remains unfavourable for the CM process. Therefore, while properly designed QD PVs exhibit a discernible CM contribution to a photocurrent, the overall improvement in power conversion efficiency is fairly small^7^. This suggests that the primary challenge in harnessing CM for practical photoconversion is the reduction of *ε*_eh_.

In the present work we tackle this challenge by exploiting not standard (spin-insensitive) but spin-exchange Coulomb interactions. In particular, recent studies of Mn-doped CdSe QDs demonstrate that energy transfer from an excited Mn ion (Mn*) to carriers residing in QD intrinsic states is extremely fast (~100 fs timescale), which allows for the ejection of a hot electron outside the dot prior to its cooling to the band edge^26,29^. The estimated energy gain rate reaches very high values of more than 10 eV ps^–1^, and as a result, it overshoots the energy loss rate by a factor of approximately seven (refs. ^26,29^). This is a dramatic departure from the standard situation when *r*_gain,ii_/*r*_loss_ < 0.3, which is expected to lead to the enhanced CM.

Here we use Mn-doped core/shell PbSe/CdSe QDs to demonstrate that spin-exchange interactions indeed open a new, highly efficient CM pathway. This pathway includes two steps: very fast spin-exchange excitation transfer from the light-harvesting CdSe shell to a Mn dopant, followed by spin-flip relaxation of Mn* accompanied by generation of two excitons in the PbSe core. We also detect weak signatures of radiative decay of Mn* leading to the formation of a PbSe-core exciton and a near-infrared (NIR) photon whose energy is defined by the difference between the energy of the Mn spin-flip transition and the PbSe-core bandgap.

## Spin-exchange Auger recombination

**Fig. 1:**
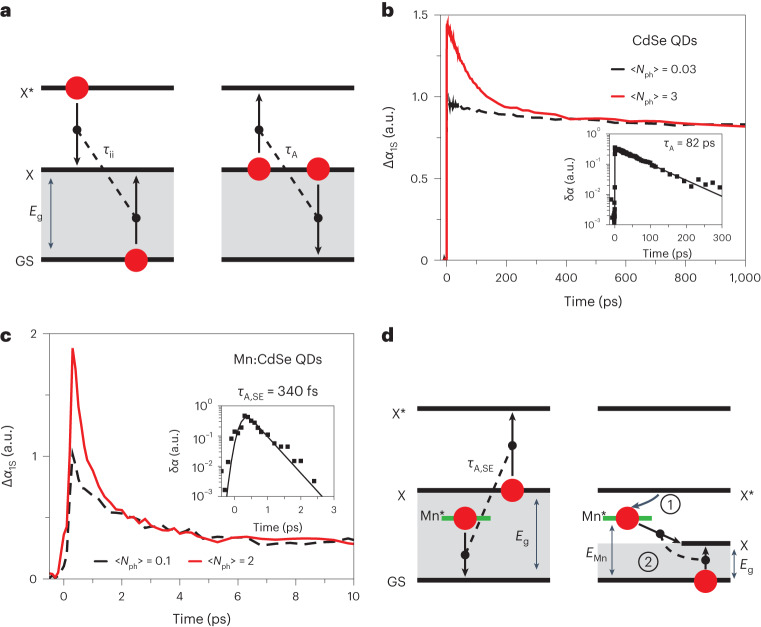
Auger recombination and impact ionization in undoped and Mn-doped QDs. **a**, Impact ionization (left) can be thought of as the inverse of Auger recombination (right); GS is the ground state, and X and X* are the band-edge and the hot-exciton states, respectively. During Auger recombination, the energy of one exciton is transferred to the other, which leads to the formation of a hot-exciton state (right). In the course of impact ionization, a hot exciton loses its kinetic energy by creating a new exciton (left). **b**, The 1S TA dynamics of the undoped CdSe QDs obtained using 2.4 eV excitation and two different pump fluences, 〈*N*_ph_〉 = 0.03 (black) and 3 (red). The higher pump intensity trace exhibits a fast initial component (~82 ps time constant) due to multi-carrier Auger recombination. The inset shows the extracted Auger dynamics obtained by subtracting tail-normalized high- and low-pump-fluence traces (δ*α* is the difference between TA signals for the different 〈*N*_ph_〉 values). **c**, The same measurements as in **b**, applied to the Mn-doped sample (〈*N*_ph_〉 = 0.1 and 2), reveal considerably faster dynamics arising from spin-exchange Auger recombination (note a 100-fold difference in the overall time spans in **c** and **b**). Based on the ‘extracted’ Auger decay (inset), the characteristic time constant is 340 fs. **d**, An ‘excitonic’ representation of spin-exchange Auger recombination (left) and spin-exchange CM (right). In the first process, the energy released during spin-flip relaxation of the excited Mn ion (Mn*), *E*_Mn_, is transferred to the QD band-edge exciton, leading to the formation of a hot exciton. During spin-exchange CM, the Mn ion excited via capture of a hot exciton (step 1) relaxes by generating two band-edge excitons (step 2). Source data

Impact ionization is often described as inverse Auger recombination (Fig. 1a). Being closely related, these two processes are characterized by the interdependent energy gain rates (*r*_gain,ii_ and *r*_gain,A_, respectively), implying that the increase in *r*_gain,A_ should translate into the increased *r*_gain,ii_. To elucidate the scale of a potential enhancement in *r*_gain,ii_ due to spin-exchange interactions, we conducted a comparative analysis of Auger decay in undoped and Mn-doped CdSe QDs with a thin protective CdS shell of approximately one semiconductor monolayer. The doped and undoped QDs had a similar overall radius of 2.2–2.4 nm. The content of internal Mn ions in the doped sample was ~1% of all cations.

To study the Auger dynamics, we apply a femtosecond transient absorption (TA) experiment to probe the evolution of the pump-induced band-edge (1S) absorption bleaching (Δ*α*_1S_; Methods). The pump photon energy (*hv*_p_) used in these measurements is 2.41 eV. Based on the TA dynamics of the undoped QDs (Fig. 1b), the biexciton Auger lifetime (*τ*_A_) is ~82 ps, which agrees with previous studies of CdSe QDs of comparable sizes^30^. Using this value and the QD bandgap of 2.13 eV (determined from the position of the 1S absorption peak; Supplementary Fig. 1), we obtain an Auger energy-gain rate due to standard, ‘direct’ Coulomb interactions of 0.026 eV ps^–1^.

The dynamics of the Mn-doped samples are dramatically different (Fig. 1c). Even at sub-single-exciton pump levels when 〈*N*_ph_〉 = 0.1 (〈*N*_ph_〉 is the average number of photons absorbed per dot per pulse), the 1S bleach decay is extremely fast (the initial 110 fs component is followed by 2.8 ps decay), which reflects the ultrafast spin-exchange energy transfer from the intrinsic QD exciton state to the Mn dopants (the faster and slower time constants correspond to the internal and surface-located dopants, respectively)^26,29^. In the Mn ground state, the spins of all five 3*d* electrons are co-aligned, which corresponds to a total spin of 5/2 (^6^A_1_ configuration)^31^. Due to energy transfer from the QD, Mn undergoes a transition to the excited 3/2 spin state wherein one of the 3*d* electron spins is flipped (Supplementary Fig. 2a depicts this process using a ‘spin-exchange’ representation^32^). Based on the pump photon energy used in these measurements (2.41 eV), excitation transfer creates the ^4^T_1_ state of the ^4^T_*m*_ manifold whose energy is *E*_Mn,T1_ = 2.1 eV. Another excited, higher-energy Mn state of relevance to the present study is ^4^T_2_ (its energy is *E*_Mn,T2_ = 2.55 eV). Its involvement will be discussed later in the context of CM measurements.

The fast subpicosecond component, which reflects the QD–Mn interactions, becomes more pronounced in the regime of multiexciton excitation (Fig. 1c). In particular, when 〈*N*_ph_〉 = 2, approximately half of the 1S bleach decays with the 340 fs characteristic time, which is a consequence of the spin-exchange Auger recombination of a hybrid state comprising an intrinsic QD exciton (X) and an excited Mn ion^29^. During this process, Mn* undergoes spin-flip relaxation to the ground state accompanied by a spin-conserving energy transfer to a QD exciton, which is promoted to a higher-energy, hot-exciton state (XMn* → X*Mn; Fig. 1d, left). In Supplementary Fig. 2b, this process is depicted using a ‘spin-exchange’ representation.

Using the measured spin-exchange Auger lifetime (*τ*_A,SE_ = 340 fs), we can estimate the energy gain rate from the ratio of *E*_Mn,T1_ and *τ*_A,SE_, which yields ~6 eV ps^–1^. This is a very considerable (more than 200-fold) enhancement versus a standard Auger process, suggesting that the energy gain rate achieved with impact ionization can also be enhanced via the involvement of spin-exchange Coulomb interactions.

## QD design for spin-exchange CM

**Fig. 2:**
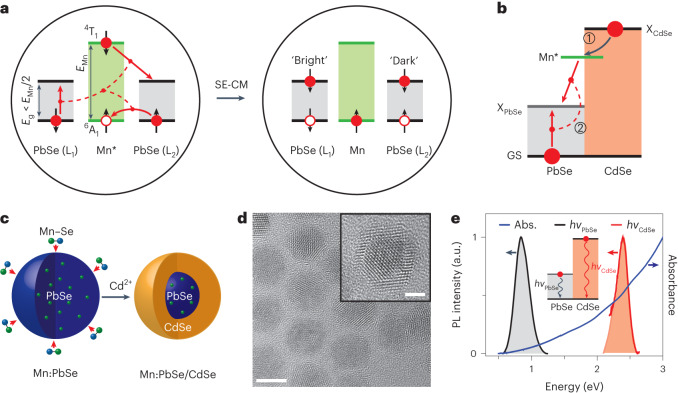
Spin-exchange CM. **a**, Spin-exchange CM (SE-CM) in Mn-doped PbSe QDs. Spin directions are shown by short, black single-sided arrows. Spin-conserving transitions are shown by red arrows. Due to spin conservation, the biexciton produced via relaxation of the excited Mn ion is a combination of a dark and a bright exciton (spins 1 and 0, respectively) in two different L valleys of PbSe (L_1_ and L_2_). The final biexciton state comprises two band-edge electrons with co-aligned spins, which is impossible in a single-valley semiconductor due to Pauli exclusion. **b**, An excitonic representation of spin-exchange CM in Mn-doped PbSe/CdSe core/shell QDs. An exciton generated in the CdSe shell (X_CdSe_) initiates the first step of spin-exchange CM, which is formation of the excited Mn state (Mn*) due to CdSe–Mn spin-exchange energy transfer (step 1). During step 2, Mn* undergoes spin-flip relaxation by creating two excitons in the PbSe core (X_PbSe_) via the spin-exchange process depicted in **a**. **c**, A schematic depiction of QD synthesis. Mn dopants are incorporated into preformed PbSe QDs via diffusion doping. The CdSe shell is formed using a controlled cation exchange reaction during which the original cations in the peripheral region of the QD are replaced with the Cd^2+^ ions. **d**, A typical TEM image of Mn-doped PbSe/CdSe core/shell QDs (scale bar, 5 nm). Inset shows a higher magnification view of an individual QD, which displays a clear core/shell structure (scale bar, 2 nm). **e**, Absorption (Abs.; blue) and dual-band PL (red and black) spectra of the Mn-doped PbSe/CdSe QDs (sample Mn-1). The NIR (black; *hv*_PbSe_ = 0.83 eV) and visible-range (red; *hv*_CdSe_ = 2.38 eV) PL bands are due to emissions from the PbSe core and the CdSe shell, respectively (inset). Source data

To enable spin-exchange CM, one needs to realize the regime wherein the excited Mn ion decays by producing two excitons without violating either energy or spin conservation. To ensure energy conservation, the Mn* energy must be at least twice the QD bandgap (Fig. 1d, right). To ensure spin conservation, the overall spin of the final biexciton must be equal to the difference in spins of the ^6^A_1_ and ^4^T_*m*_ states (Δ*S*_Mn_ = 5/2 − 3/2 = 1). These two conditions can, in principle, be satisfied with PbSe QDs, which have been extensively studied in the context of ordinary CM^5–7,9,10,18–20^. PbSe is a narrow-gap material with a bulk bandgap of 0.27 eV. Using properly sized QDs, *E*_g_ can be tuned to be a desired fraction of the Mn* energy (for example, *E*_Mn,T1_/2 or less). Furthermore, the PbSe band structure features four equivalent L valleys comprising band-edge excitons with spins *S* = 0 and 1 (‘bright’ and ‘dark’ excitons, respectively)^33,34^. In this case, the spin conservation requirement can be met if Mn* decays to create a biexciton composed of, for example, a dark and a bright band-edge exciton. These two excitons must reside in two different L valleys as Pauli exclusion does not allow same-spin electrons or holes to occupy the same band-edge level (Fig. 2a).

One potential problem in the above scheme is weak exchange coupling in Mn-doped PbSe QDs (hole and electron exchange parameters are, respectively, *N*_0_*β* = −0.08 eV and *N*_0_*α* = 0.02 eV (refs. ^35,36^); here, *α* and *β* are the corresponding exchange constants and *N*_0_ is the number of cations per unit semiconductor volume), which may not allow for efficient capture of a hot exciton by the Mn ion (process 1 in Fig. 1d, right). To mitigate this potential complication, we have chosen an alternative system, which is core/shell PbSe/CdSe QDs, the structures previously studied in the context of ordinary CM^23^. We hypothesize that in Mn-doped PbSe/CdSe QDs, the CdSe shell will serve as both a light-harvesting antenna and a highly efficient sensitizer of Mn ions (Fig. 2b). Indeed, as evident in Fig. 1c, in Mn:CdSe QDs, exciton transfer to a Mn ion occurs on the ultrafast (~100 fs) timescale, which is a consequence of the large strength of the Mn–CdSe exchange coupling (*N*_0_*β* = −1.27 eV and *N*_0_*α* = 0.23 eV)^37,38^. This will ensure a high efficiency of the first step of spin-exchange CM, the hot-exciton capture by the Mn dopants (*hv*_p_ → X*_CdSe_ → Mn*; Fig. 2b). In the second step, Mn* relaxes to the ground ^6^A_1_ state by generating a bright and a dark exciton in the PbSe core (X_PbSe,0_ and X_PbSe,1_, respectively), that is, Mn* → X_PbSe,0_ + X_PbSe,1_ (Fig. 2a,b). The second step should not be hampered by weak Mn–PbSe coupling, as a competing channel of Mn* relaxation via a direct ^4^T_1_−^6^A_1_ spin-flip transition is very slow and occurs on millisecond timescales^39,40^.

In the proposed scheme, the Mn dopant serves as a spin-exchange mediator, which enables the conversion of an original exciton generated in the CdSe shell into two lower-energy excitations in the PbSe core. This motif of ‘two for the price of one’ is somewhat similar to one explored recently in the context of ‘quantum cutting’ in Yb-doped perovskite QDs^41,42^ wherein an original QD exciton is converted into two excited impurity ions whose deactivation occurs via emission of two NIR photons.

## Synthesis and characterization of Mn-doped PbSe/CdSe QDs

**Extended Data Fig. 1:**
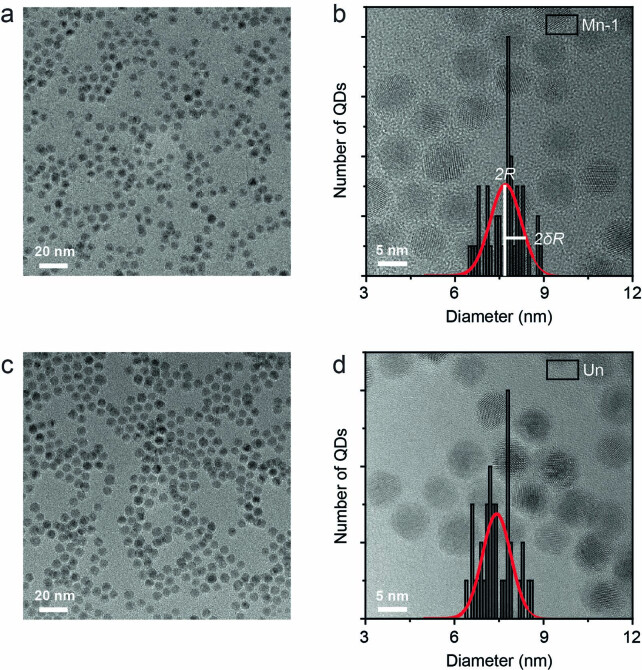
Transmission electron microscopy (TEM) measurements of undoped and Mn-doped PbSe/CdSe QDs. The TEM images and size distributions of (**a, b**) the Mn-doped (sample ‘Mn-1’) and (**c, d**) the reference, undoped (sample ‘Un’) PbSe/CdSe core/shell QDs. Red curves are Gaussian fits of size distributions shown by black vertical bars. *R* is the average overall radius of the QDs and *δR* is the standard deviation. Mn-doped QDs have a slightly larger size than undoped QDs. This is due to addition of Mn-Se units during the diffusion doping process, which leads to the increase of the overall size of the QDs.

**Extended Data Fig. 2:**
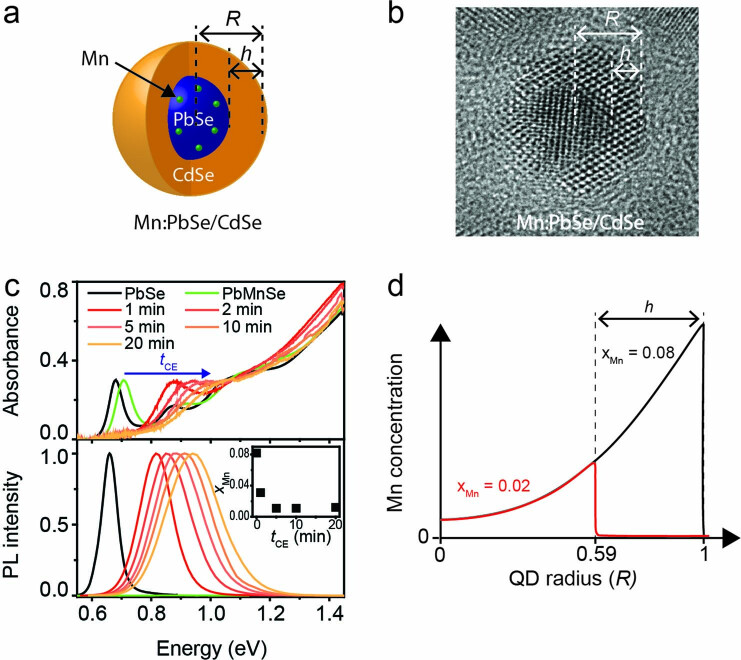
Mn-doped PbSe/CdSe core/shell QDs. **a**, A schematic depiction of a Mn-doped PbSe/CdSe QD. **b**, An exemplary TEM image of a QD from one of the Mn-doped PbSe/CdSe QD samples (sample Mn-1). The PbSe core radius (*r*) is 2.3 nm, the CdSe shell thickness (*h*) is 1.6 nm, and the overall QD radius (*R*) is 3.9 nm. **c**, The absorption (top) and emission (bottom) spectra of the Mn-doped PbSe/CdSe QDs as a function of duration of a cation exchange reaction (*t*_CE_) leading to formation of the CdSe shell (red and orange traces). The spectra of the original undoped PbSe QDs are shown in black. The spectra of Mn-doped PbSe QDs before cation exchange are shown in green. After doping with Mn, the PL of the PbSe QDs is completely quenched. It is recovered after the formation of the CdSe shell. The progressive increase of the CdSe-shell thickness leads to the shrinkage of the PbSe core, which manifests as a blue shift of the PL spectrum and the band-edge absorption feature. Inset: Mn content as a function of *t*_CE_. **d**, Modeling of diffusion doping of PbSe QDs using Fick’s 2^nd^ law of diffusion leads to a Mn distribution shown by the black line. The diffusion parameters are selected so as to yield the total content of Mn ions of 8% (defined by the area under the black trace), which corresponds to the experimental situation in the case of sample Mn-1. Then, we assume that during cation exchange, Cd^2+^ replaces the original cations within the shell region. The resulting distribution of Mn ions is given by the red trace. Based on the area under this trace, the Mn content is reduced to 2%, which is close to the experimental value of 1.6%. This analysis suggests that the distribution of the Mn ions is peaked at the core/shell interface and gradually decays towards the PbSe core centre. Source data

To prepare Mn-doped PbSe/CdSe QDs, we applied a diffusion doping approach (Methods)^43,44^. Briefly, we fabricated PbSe QDs using procedures described in the literature^45^ and then reacted them with Mn–acetate, which led to the incorporation of magnetic impurities into the QD surface layer, followed by their diffusion into the QD interior. Then we used a standard cation exchange reaction to introduce Cd into a peripheral region of the QD^23,45^. This resulted in the formation of a CdSe shell of a controlled thickness accompanied by the shrinking of the PbSe core (Fig. 2c and Extended Data Fig. 1). Based on our modelling of diffusion doping followed by cation exchange (Extended Data Fig. 2), the distribution of Mn ions peaks at the CdSe–PbSe interface and gradually falls off towards the centre of the PbSe core. As a result, Mn ions exhibit good exchange coupling to both an electron and a hole, as both carriers are preferentially core localized^23,46^.

Most of the spectroscopic results reported in the following were collected for a QD sample labelled Mn-1. Based on transmission electron microscopy (TEM) measurements (Fig. 2d), it contains highly monodisperse, nearly spherical particles with a well-defined core/shell structure. The overall QD radius (*R*) and the shell thickness (*h*) are 3.9 nm and 1.6 nm, respectively. These parameters yield the aspect ratio *ρ* = *h*/*R* = 0.41. The Mn content is 1.6% of all cations, which corresponds to ~67 ions per dot on average.

**Extended Data Fig. 3:**
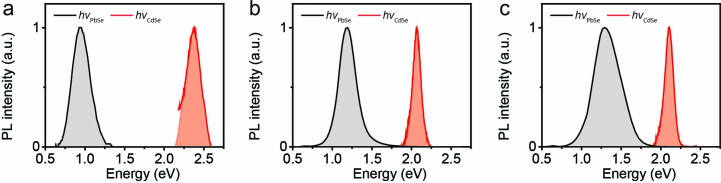
Dual-band emission spectra of Mn-doped PbSe/CdSe QDs. PL spectra of the Mn-doped samples: (**a**) Mn-2, (**b**) Mn-3, (**c**) Mn-4. All samples show dual-band emission originating from the core (black) and the shell (red) of the PbSe/CdSe QDs. Source data

Based on the photoluminescence (PL) spectra (Fig. 2e), the bandgap of these QDs (defined by the spacing between the conduction and valence band-edge states of the PbSe core) is 0.83 eV. To elucidate the effect of *E*_g_ on the studied spin-exchange processes, we prepared three additional Mn-doped samples with *E*_g_ = 0.91, 1.18 and 1.27 eV (samples Mn-2, Mn-3 and Mn-4, respectively). Furthermore, we synthesized an undoped reference sample whose dimensions and spectroscopic characteristics closely matched those of the Mn-1 sample. Detailed spectroscopic and TEM characterizations of these additional samples can be found in Extended Data Fig. 3, Supplementary Figs. 3–5 and Extended Data Table 1.

As was previously observed for high-aspect-ratio, undoped PbSe/CdSe QDs^23^, the Mn-1 sample exhibits dual-band emission due to band-edge transitions associated with the PbSe and CdSe QD components, whose energies are *hv*_PbSe_ = 0.83 eV and *hv*_CdSe_ = 2.38 eV (Fig. 2e). The lowest of these energies defines the QD bandgap: *E*_g_ = *hv*_PbSe_. The dual-band spectra are also observed for three other studied Mn-doped samples (Extended Data Fig. 3).

## CM measurements

In our experimental studies of spin-exchange CM, we monitor the dynamics of the band-edge PbSe-core states using transient PL and TA spectroscopies. In particular, we use a distinctive fast multiexciton Auger decay component to detect CM and to evaluate its efficiency^4,5,20,47–49^.

**Fig. 3:**
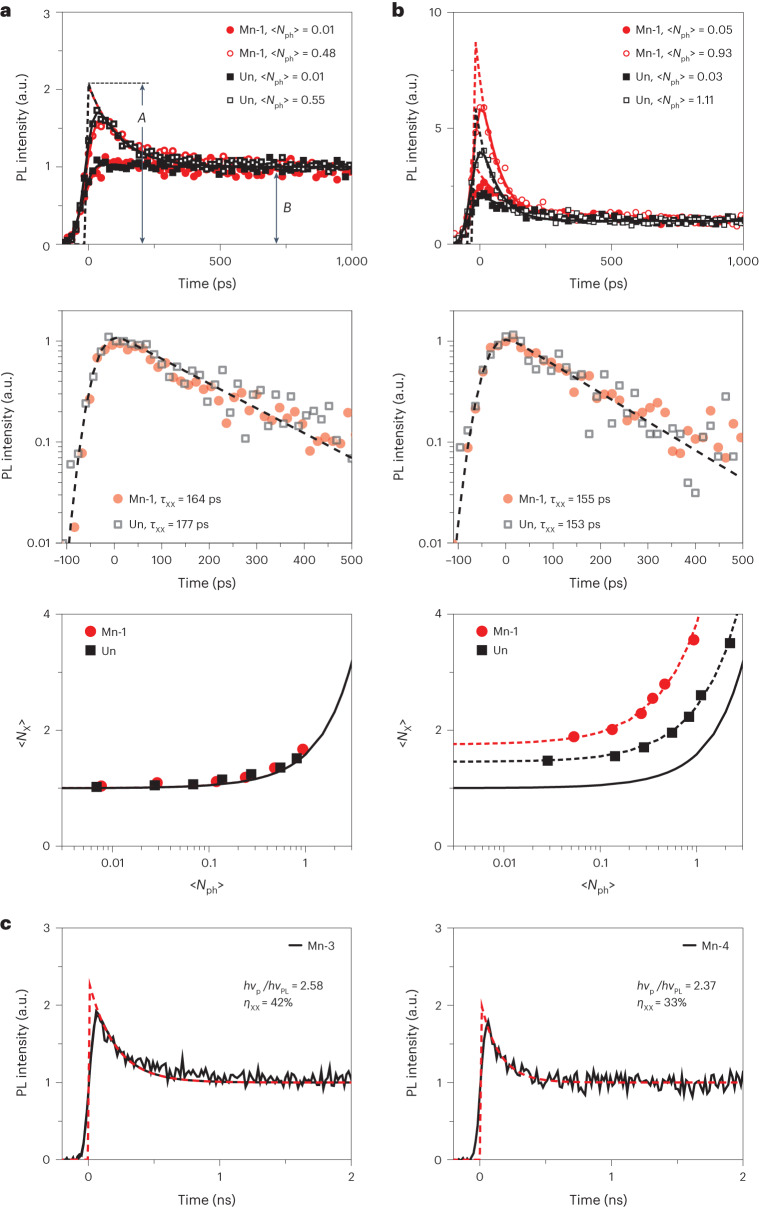
CM measurements of undoped and Mn-doped PbSe/CdSe core/shell QDs. **a**, Time-resolved intensity of the NIR PbSe-core emission for the Mn-doped (Mn-1, red) and the undoped (Un, black) QDs at low (solid symbols) and high (open symbols) pump fluences (top panel; 〈*N*_ph_〉 = 0.01 and ~0.5, respectively). These dynamics were measured using pump photons with a sub-CM-threshold energy (*hv*_p_ = 1.20 eV and 1.55 eV for the doped and the undoped samples, respectively). The traces are normalized so as to match the long-time tails. The symbols are raw data, and the lines are traces obtained via a deconvolution procedure to account for the 58 ps IRF. The higher pump-intensity traces develop a fast initial component that is absent in the dynamics recorded using the low pump level. The fast PL component (middle panel; symbols; isolated via subtraction of high- and low-pump-intensity traces) is due to the Auger decay of bi-excitons. It exhibits exponential dynamics with time constant *τ*_XX_ ≈ 170 ps (dashed line), which is the same for the doped and undoped samples. The exciton multiplicity (〈*N*_X_〉) calculated from the *A*/*B* ratio of the ‘deconvolved’ PL traces as a function of 〈*N*_ph_〉 (symbols; bottom panel) is fitted using the Poisson statistics of photon absorption events (line; bottom panel). **b**, A similar set of data but obtained using 3.1 eV excitation, which is above the CM threshold. As distinct from **a**, even the lowest intensity traces exhibit a fast bi-excitonic component (top) whose time constant (middle) is similar to that obtained using low photon excitation. Another distinction is a strong deviation of the measured 〈*N*_X_〉 (symbols; bottom panel) from the Poisson dependence (solid black line; bottom panel). In particular, the low-〈*N*_ph_〉 limit is greater than 1, a typical signature of CM. Based on these data, multiexciton yields are 48% and 75% for the undoped and doped samples, respectively. **c**, The PbSe-core NIR PL dynamics of doped samples Mn-3 (left) and Mn-4 (right) also exhibit a pronounced bi-excitonic component despite the use of low, sub-single-exciton pump levels (〈*N*_ph_〉 < 0.1). This points towards highly efficient CM. *hv*_PL_ is the PL detection energy, which defines the bandgap of the QDs probed in the CM measurements. Source data

First, we quantify biexciton Auger lifetimes in our core/shell samples via pump-intensity-dependent PL measurements conducted with pump photons of low energy (*hv*_p_ < 2*E*_g_), for which CM is not energetically possible. In Fig. 3a (top), we display the PL dynamics for the Mn-1 sample (red symbols) and the reference undoped QDs (black symbols), obtained using *hv*_p_ = 1.2 eV and 1.5 eV, respectively. In these measurements, Auger recombination manifests as a fast initial component that develops when 〈*N*_ph_〉 ≈ 1 (open symbols in Fig. 3a, top). Since its lifetime is comparable to the temporal resolution of our PL measurements (the width of the instrument response function, IRF, is 58 ps; Supplementary Fig. 6), we use the deconvolution with the IRF to obtain a ‘true’ shape of the PL time transients (lines in Fig. 3a). Based on the deconvolved dynamics, in both the doped and the undoped sample, the biexciton Auger lifetime is ~170 ps (Fig. 3a, middle), which is consistent with previous measurements of undoped PbSe/CdSe QDs^23^.

Further evidence that the initial fast signal is due to bi-excitons is obtained from the analysis of the average exciton multiplicity (〈*N*_X_〉; the number of excitons per dot in a subensemble of photoexcited QDs^4^). This quantity can be determined from the ratio of the early (*A*) and late (*B*) time PL signals (Fig. 3a, top) using 〈*N*_X_〉 = (*A*/*B* + 2)/3 (refs. ^47–49^). In the case of standard Poisson statistics of photon absorption events, 〈*N*_X_〉 = 〈*N*〉(1 − e^−〈*N*〉^)^−1^, where 〈*N*〉 is the average per-dot number of photogenerated excitons, which without CM is equal to 〈*N*_ph_〉. For both the doped and undoped samples, the measured 〈*N*_X_〉 closely follows the Poisson dependence (Fig. 3a, bottom), confirming that the fast initial PL component is due to multi-excitons generated via absorption of multiple photons from the same pump pulse.

Next, we conduct the same measurements using higher-energy 3.1 eV pump photons (Fig. 3b). In this case, the fast bi-excitonic component is present even at the lowest pump levels (〈*N*_ph_〉 ≤ 0.05), a typical signature of CM. Furthermore, the measured exciton multiplicity (symbols in Fig. 3b, bottom) strongly deviates from the Poisson dependence (black solid line in Fig. 3b, bottom), again as expected for CM when bi-excitons are generated by single photons^47–49^.

The value of 〈*N*_X_〉 in the limit of vanishingly small pump powers yields *Q*_eh_. Based on a linear extrapolation of 〈*N*_X_〉 measured for the reference sample, *Q*_eh_ = 1.48 = 148%. This corresponds to the biexciton yield (*η*_XX_ = *Q*_eh_ − 1) of 48%, in line with previous measurements of PbSe/CdSe QDs for the similar *hv*_p_/*E*_g_ ratio of 3.8 (ref. ^23^). Interestingly, for the doped Mn-1 sample, *η*_XX_ is increased to 75% (*Q*_eh_ = 175%), despite a slightly lower value of *hv*_p_/*E*_g_ (3.7). The CM enhancement is also observed for other QD sizes with a larger bandgap (Fig. 3c). For example, for the Mn-doped QDs with *E*_g_ = 1.2 eV (sample Mn-3; Fig. 3c, left), *η*_XX_ = 42%. This is a factor of approximately three higher than the *η*_XX_ value previously measured for the similarly sized, undoped PbSe/CdSe QDs^23^.

**Fig. 4:**
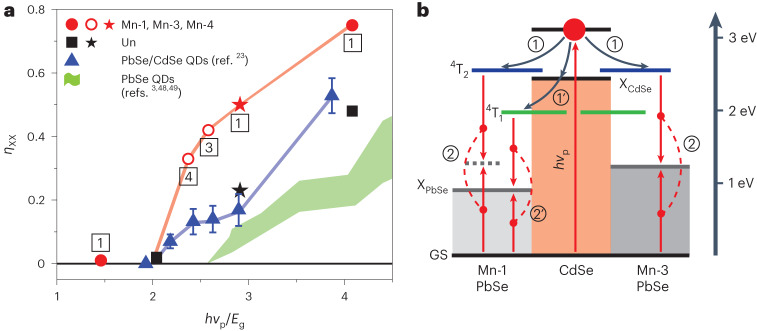
CM efficiencies in undoped and Mn-doped QDs. **a**, Multiexciton yields (*η*_XX_) as a function of photon energy normalized by the bandgap (*hv*_p_/*E*_g_). The data from the present study are shown by red (Mn-doped QDs) and black (undoped QDs) symbols; circles and squares are the PL measurements, and stars are the TA measurements. Labels 1 to 4 are sample numbers for the Mn-doped QDs. The PL and TA data were obtained using *hv*_p_ = 3.1 eV and 2.41 eV, respectively. The blue triangles show the CM measurements of undoped PbSe/CdSe QDs from ref. ^23^ (the error bars are from the same work). CM efficiencies of core-only PbSe QDs are schematically shown by green shading (refs. ^3,48^^,^^49^). **b**, An excitonic representation of spin-exchange CM for samples Mn-1 and Mn-3 in the case of 3.1 eV excitation. This process occurs via activation of the Mn ion via exciton transfer from the CdSe shell (step 1 or 1′) followed by Mn* relaxation, which creates a biexciton in the PbSe core (step 2 or 2′). In the case of 3.1 eV excitation, the energy of a photogenerated hot exciton is sufficiently high to excite both the ^4^T_2_ and ^4^T_1_ states of the Mn ion (the ^4^T_1_ state can also be excited via capture of a band-edge exciton following hot-exciton cooling). In sample Mn-1, spin-exchange CM can be driven by both the ^4^T_2_−^6^A_1_ and ^4^T_1_−^6^A_1_ spin-flip transitions. However, in sample Mn-3, which has a higher bandgap, spin-exchange CM can be driven only by the higher-energy ^4^T_2_−^6^A_1_ transition. Due to the larger number of spin-exchange CM pathways, sample Mn-1 shows a higher CM efficiency than sample Mn-3 (**a**). Source data

**Extended Data Fig. 4:**
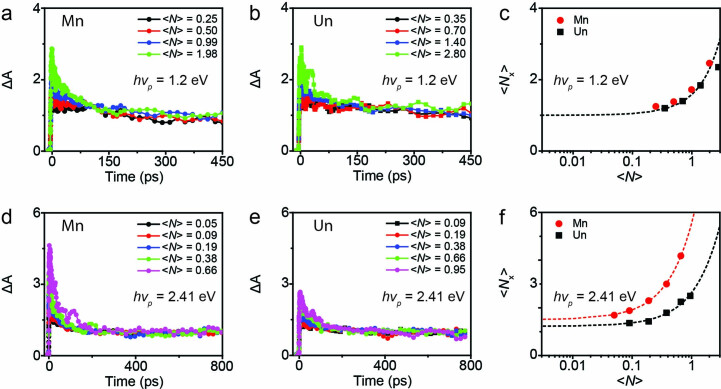
Transient absorption (TA) measurements of carrier multiplication (CM) in undoped and Mn-doped PbSe/CdSe QDs. Pump-fluence-dependent femtosecond TA measurements of band-edge bleach dynamics in Mn-doped (sample Mn-1; **a, c, d, f**) and undoped (sample Un; **b, c, e, f**) PbSe/CdSe QDs probed at *hv*_probe_ = 0.84 eV (ΔA is the pump-induced change in sample absorbance). The measured transients are normalized so as to match the long-time tails. These measurements were conducted using pump photon energies *hv*_p_ = 1.2 eV (**a**–**c**) and 2.41 eV (**d**–**f**), which corresponded to excitation below and above the CM threshold, respectively. In both cases, TA dynamics develop a fast Auger decay component at higher pump levels, which is consistent with generation of multiexcitons. However, only for the 2.41-eV excitation, the fast Auger component persists in the limit of sub-single-exciton pump levels, which is a signature of CM. In the case of *hv*_p_ = 1.2 eV, for both doped and undoped samples (**c**; red and black symbols respectively), the exciton multiplicity (〈*N*_X_〉) derived from the analysis of the early- and late-time TA amplitudes tends to 1 in the limit of low pump fluences, indicating no CM. However, the same analysis for the case of *hv*_p_ = 2.41 eV indicates 〈*N*_X_〉 of 1.23 and 1.5 for the undoped (**f**; black squares) and doped (**f**; red circles) samples, respectively. This is a signature of CM. The biexciton yield in the Mn-doped sample (*η*_XX_ = 50%) is more than twice as large as in the undoped sample (*η*_XX_ = 23%). Dashed lines in **‘c’** and **‘f’** are linear fits to the data. Source data

To verify the results of the PL measurements, we conducted TA studies of the Mn-doped and undoped samples using 1.2 eV and 2.41 eV pump photons that correspond to an excitation below and above the CM threshold, respectively (Extended Data Fig. 4). For the Mn-1 sample, these measurements reveal more than twofold CM enhancement versus the undoped QDs (Fig. 4a, red and black stars, respectively), which is comparable to the enhancement observed in the PL measurements.

In Fig. 4a, we present a plot of *η*_XX_ versus *hv*_p_/*E*_g_, which summarizes the results of our PL (circles and squares) and TA (stars) measurements of doped (red) and undoped (black) core/shell samples (also Supplementary Table 1). These data are compared to the literature CM results for the undoped PbSe/CdSe QDs^23^ (triangles) and core-only PbSe QDs^48,49^ (green shading). All Mn-doped samples show a considerable CM boost compared to not only PbSe QDs but also core/shell QDs. Importantly, the enhancement is especially large at energies near the nominal CM threshold (*hv*_th,nom_ = 2*E*_g_). For example, for *hv*_p_ ≈ 2.6*E*_g_, the Mn-doped samples show *η*_XX_ ≈ 42%, while CM is completely absent in PbSe QDs and *η*_XX_ is only ~14% in the undoped PbSe/CdSe QDs^23^.

For deeper insight into the mechanism of spin-exchange CM, we analyse the energies of the relevant QD and Mn-ion transitions (Fig. 4b). The minimal photon energy required to drive ordinary CM is 2*E*_g_. In the case of spin-exchange CM, an additional requirement is that 2*E*_g_ must be lower than the energy of at least one of the spin-flip transitions of the Mn ion. Based on the latter condition, sample Mn-4 (2*E*_g_ = 2.54 eV) is right at the threshold of spin-exchange CM (*E*_Mn,T2_ = 2.55 eV is just slightly higher than 2*E*_g_). This might explain the sharp, stepwise increase in the CM yield associated with this sample in the plot of Fig. 4a. We observe a further modest increase in *η*_XX_ for sample Mn-3, which likely occurs due to the reduction in 2*E*_g_ (to 2.36 eV) leading to the increased ‘energetic driving force’ for spin-exchange CM (defined by *E*_Mn,T2_ − 2*E*_g_).

As illustrated in Fig. 4b and Supplementary Fig. 7, the CM yield also increases with the increasing number of accessible Mn* spin-flip transitions capable of driving CM. This, in particular, might contribute to the growth of *η*_XX_ observed for sample Mn-1 when *hv*_p_ changes from 2.41 eV to 3.1 eV (Fig. 4a; the red star and the red solid circle, respectively). The first of these energies can access only the *E*_Mn,T1_ transition, while the second is sufficiently high to excite both the *E*_Mn,T1_ and *E*_Mn,T2_ transitions, and both of them can instigate CM.

A further indication of the enhancement of the CM process due to spin-exchange interactions is provided by the analysis of the e–h pair creation energy. The upper bound of this quantity can be estimated from the ratio of Δ*E* = *hv*_p_ − *hv*_th,nom_ and *η*_XX_: *ε*_eh_ ≤ Δ*E*/*η*_XX_ (refs. ^48,49^). Applying this expression to samples Mn-1 and Mn-4 (Supplementary Fig. 8), we obtain *ε*_eh_ ≈ 1.8*E*_g_ and 1.3*E*_g_. In the case of Mn-4, the nominal CM threshold (2*E*_g_) is close to *E*_Mn,T2_, which helps reduce energy losses during the Mn*-to-2X_PbSe_ transition and thereby helps minimize *ε*_eh_. Importantly, the *ε*_eh_ value realized with Mn-doped samples is up to two times lower than that for the undoped core/shell QDs (*ε*_eh_ ≥ 2.6*E*_g_).

## Radiative spin-exchange Mn-to-PbSe core coupling

**Fig. 5:**
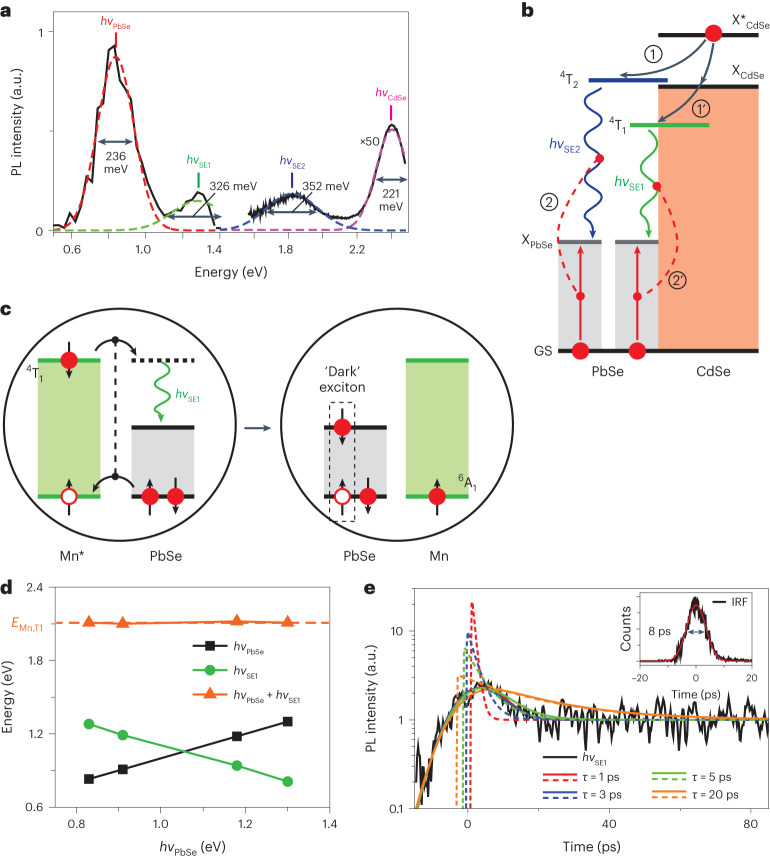
Observations of radiative spin-exchange Mn–PbSe-core coupling. **a**, The PL spectrum of Mn-doped QDs (sample Mn-1) spanning from a NIR to a visible spectral range. The lower-energy part of the spectrum (~0.5 eV to ~1.4 eV) is instantaneous PL measured using a superconducting single-photon detector (SSPD) at 10 ns after excitation with 1.55 eV, 50 fs pump pulses. Using a time-resolved SSPD technique, we are able to observe simultaneously a long-lived *hv*_PbSe_ band and a short-lived *hv*_SE1_ feature. The higher-spectral-energy PL (>1.5 eV), which comprises short-lived *hv*_CdSe_ and *hv*_SE2_ features, is time-integrated emission measured with a standard Si detector using 3.1 eV excitation. The observed PL features are fitted to Gaussian bands whose widths (defined as a full-width at half-maximum) are indicated in the figure. **b**, An excitonic representation of radiative channels leading to the *hv*_SE1_ and *hv*_SE2_ emission features. Steps 1′ and 1 are excitation of the ^4^T_1_ and ^4^T_2_ states of the Mn ion via exciton transfer from the CdSe shell. Step 2 or 2′ is Mn* decay to produce a photon and a PbSe-core exciton. **c**, A more rigorous spin-exchange depiction of the emission pathway leading to the *hv*_SE1_ PL features. Due to spin conservation, decay of the ^4^T_1_ state produces a spin-1 dark PbSe core exciton. **d**, Spectral energies *hv*_PbSe_ (black squares) and *hv*_SE1_ (green circles), and their sum (*hv*_PbSe_ + *hv*_SE1_; orange triangles) as a function of *hv*_PbSe_ (based on the measurements of samples Mn-1 to Mn-4). **e**, Streak-camera measurements of the dynamics of the *hv*_SE1_ PL band (black trace) reveal a fast, resolution-limited decay component. Based on the deconvolution using the measured 8 ps IRF (the black line in the inset; the red line is a Gaussian fit; the grey arrow shows the IRF full width at half maximum), we determine that the relaxation time constant of the *hv*_SE1_ PL feature (*τ*_SE1_) is 3 ps (blue) or shorter (red; *τ*_SE1_ = 1 ps*)*. The use of longer time constants (5 ps and 20 ps; green and orange traces, respectively) leads to an appreciable deviation of the modelling from the measurement. The deconvolution procedure also indicates that the fast decay component is responsible for more than 90% of the overall PL signal. The dashed and the solid lines are the model traces before and after convolution with the IRF, respectively. Source data

**Extended Data Fig. 5:**
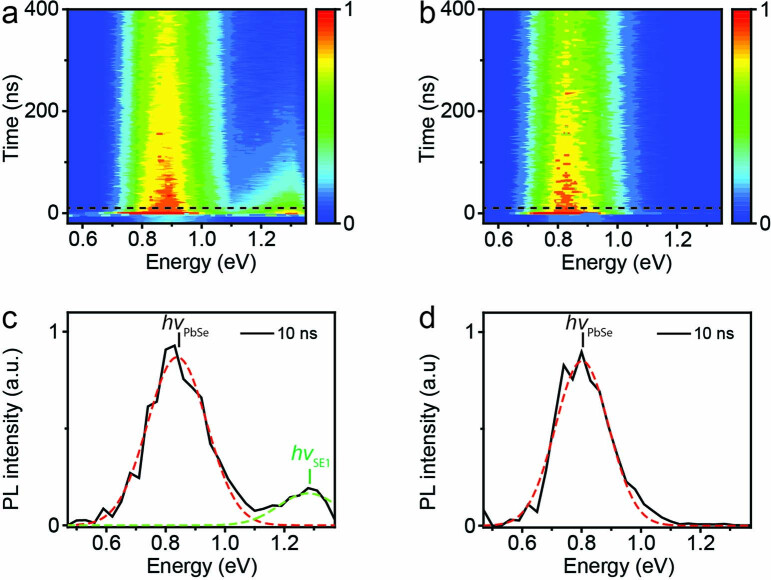
Transient photoluminescence (PL) measurements of the undoped and Mn-doped PbSe/CdSe QDs. Two-dimensional plots of time- and spectrally-resolved PL of Mn-doped QDs (sample Mn-1) (**a**) and reference, undoped QDs (**b**). These data were collected using excitation with *hv*_p_ = 3.1 eV and 〈*N*_ph_〉 < 0.1. In addition to the band-edge NIR emission from the core (*hv*_PbSe_ = 0.83 eV), the Mn-doped sample exhibits a higher-energy NIR band at 1.28 eV (*hv*_SE_), which is absent for the undoped sample. This band emerges due to radiative spin-flip relaxation of the excited Mn ion which produces a NIR photon and a PbSe-core-based exciton (see main article). Instantaneous emission spectra of the doped (**c**) and the undoped (**d**) sample obtained from **‘a’** and **‘b’**, respectively, by ‘slicing’ the two-dimensional plots at 10 ns. These spectra have been corrected for energy-dependent sensitivity of the detection system used in the measurements. The experimental spectra are fitted to Gaussian bands whose centres yield the positions of the PbSe core emission (*hv*_PbSe_, red) and the spin-exchange band (*hv*_SE1_, green). The Mn-doped QDs show both the *hv*_PbSe_ and *hv*_SE1_ features, whereas the undoped QDs exhibit only the *hv*_PbSe_ band. Source data

A closer inspection of PL spectra of the Mn-doped PbSe/CdSe QDs reveals two weak NIR bands located between the core-related and shell-related emission peaks (Fig. 5a and Extended Data Fig. 5). These features are not present in the PL of the undoped samples and, therefore, are likely associated with emission pathways involving Mn dopants. Interestingly, their energies (*hv*_SE1_ and *hv*_SE2_ for the lower- and higher-energy bands, respectively are close to the energy difference between the *E*_Mn,T1_ (*E*_Mn,T2_) transition and the PbSe bandgap (*Δ*_T1,2_ = *E*_Mn,T1,2_ − *E*_g_). For example, in the case of the Mn-1 sample, *hv*_SE1_ = 1.28 eV and *hv*_SE2_ = 1.82 eV, while *Δ*_T1_ = 1.27 eV and *Δ*_T2_ = 1.72 eV. The close correspondence between *hv*_SE1,2_ and *Δ*_T1,2_ suggests that the NIR features arise from the radiative spin-exchange transitions whereby the *E*_Mn,T1_ and *E*_Mn,T2_ states decay by generating a photon and a dark PbSe-core band-edge exciton: Mn*_T1,2_ → *hv*_SE1,2_ + X_PbSe,1_ (Fig. 5b,c). One might expect that the spin-exchange channel also produces excited PbSe-core states. Such additional radiative pathways are likely responsible for the increased broadening of the *hv*_SE1,2_ bands (330–350 meV) compared to that of the *hv*_PbSe_ feature (236 meV; Fig. 5a).

Three other Mn-doped core/shell samples also exhibit spin-exchange NIR bands whose energies are linked to the difference of the energy of the excited Mn ion and the PbSe-core bandgap (Fig. 5d and Supplementary Fig. 9). Interestingly, in samples Mn-3 and Mn-4, for which *hv*_PbSe_ is greater than *E*_Mn,T1_/2, the *hv*_SE1_ feature is located at lower energies than the *hv*_PbSe_ band, which is opposite to the situation for the two other doped samples with a smaller bandgap.

**Extended Data Fig. 6:**
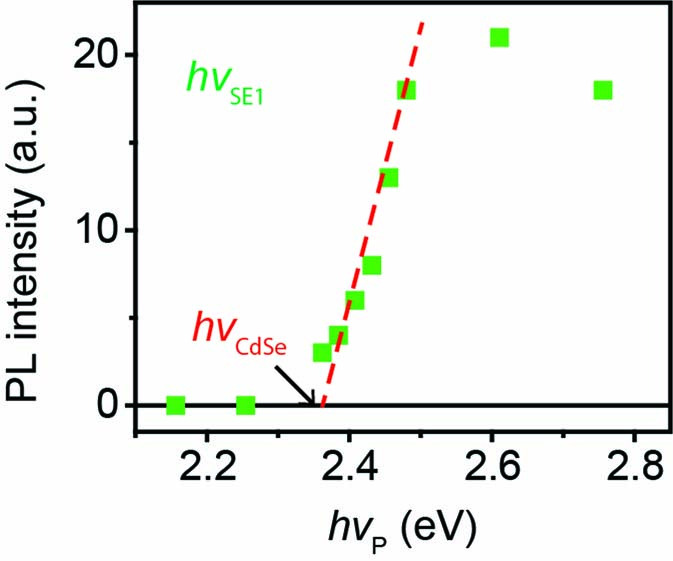
The PL excitation spectrum of the spin-exchange *hv*_SE1_ band. The intensity of the *hv*_SE1_ PL band (sample Mn-1) as a function of *hv*_p_ for constant excitation level 〈*N*_ph_〉 < 1 (green squares). This dependence shows a sharp onset at ~2.36 eV (dashed red line), which is close to the energy of the CdSe-shell-based exciton (*hv*_CdSe_ = 2.38 eV). Source data

The PL excitation measurements of the *hv*_SE1_ band indicate that it is activated via photoexcitation of the CdSe shell (Extended Data Fig. 6). This substantiates our original concern that the Mn–PbSe exchange coupling may not be sufficiently strong to ensure efficient capture of a hot exciton by the Mn ion, which motivated the use of core/shell PbSe/CdSe structures instead of core-only PbSe QDs.

## Spin-exchange CM timescale

While being clearly discernible in appropriately scaled PL spectra, the *hv*_SE1_ and *hv*_SE2_ features are very weak, and their relative PL quantum yield versus the PbSe emission is less than 4%. This implies that Mn* de-excitation occurs primarily non-radiatively via spin-exchange CM (Fig. 2a,b). Hence, the dynamics of the *hv*_SE1_ (or *hv*_SE2_) band can be used to quantify the timescale of the spin-exchange CM process.

To monitor the dynamics of the *hv*_SE1_ band, we use a NIR streak camera with 8 ps temporal resolution (Methods). An example of these measurements is displayed in Fig. 5e (black line). The recorded trace exhibits a fast, resolution-limited decay whose amplitude is ~150% of the long-time slow background. To model the initial fast relaxation, we use a convolution of single exponential decay (dashed lines in Fig. 5e) with the 8 ps IRF (inset of Fig. 5e). Using a decay constant of 3 ps or shorter, we can accurately reproduce the measurement (solid blue and red traces in Fig. 5e). This suggests that the spin-exchange CM timescale is very short (≤3 ps), which explains the high efficiency of this process.

## Discussion

To summarize, we demonstrate Mn-doped core/shell PbSe/CdSe QDs wherein magnetic ions act as highly effective mediators of spin-exchange interactions between CdSe and PbSe QD components. This opens a new CM pathway whereby the excited Mn ion undergoes spin-flip relaxation accompanied by the generation of two core-based excitons. We also detect signatures of radiative spin-exchange Mn–PbSe-core coupling that manifests as weak NIR emission features residing between the core and shell emission bands. Due to the spin-exchange contribution to the CM process, Mn-doped QDs exhibit a considerable reduction in the e–h pair creation energy compared to the undoped structures, which translates into an up-to-threefold enhancement of the CM yield at near-CM-threshold photon energies.

The observed effect is expected to lead to an appreciable increase in the power conversion efficiency of PV devices (Supplementary Section 1). It can also be exploited in solar photochemistry. The unique feature of spin-exchange CM is its ability to generate two e–h pairs colocalized in both the time and spatial domains. This could be especially useful in the case of multi-step, multi-electron/hole chemical reactions, in which one of the rate-limiting factors is a ‘wait time’ between sequential reduction (oxidation) steps. An overall conclusion of our studies is that the use of spin-exchange interactions represents a viable approach for boosting the efficiency of CM to levels that can make this phenomenon of relevance to practical photoconversion in PVs and photoinduced chemistry.

## Methods

### Materials

Lead(II) oxide (PbO, Alfa Aesar, 99.99%), selenium shot (Se, Alfa Aesar, 99.999%), selenium powder (Se, Alfar Aesar, 200 mesh, 99.999%), manganese(II) acetate tetrahydrate (Mn(ac)_2_·4H_2_O, Strem, 99.999%), cadmium oxide (CdO, Aldrich, 99.99%), oleic acid (OAc, Aldrich, 90%), oleylamine (OAm, Aldrich, 70%), 1-octadecene (ODE, Aldrich, 90%), trioctylphosphine (TOP, Aldrich, 97%), diisobutylphosphine (DIP, Strem, 97%), diphenylphosphine (DPP, Aldrich, 98%) and tetrachloroethylene (TCE, Aldrich, 99%) were used as received without additional purification. All syntheses of QDs were performed under inert conditions using standard Schlenk-line and glove box techniques.

### Synthesis of PbSe QDs

PbSe QDs were synthesized using reported methods with modification^45^. In a typical procedure, 2 M Se precursor solution in TOP (TOP-Se) was prepared by dissolving Se shot (7.896 g) in TOP (50 ml) and by stirring for 20 h in a glove box. PbO (892.8 mg), OAc (4 ml) and ODE (10 ml) were loaded in a three-neck flask and degassed at 120 °C under vacuum for 1 h. The temperature was increased to 220 °C under a N_2_ atmosphere, and a solution containing 2 M TOP-Se (1 ml), TOP (2 ml) and DIP (0.1 ml) was swiftly injected into the flask. The reaction was kept at 200 °C for 10 min and then quenched by removing the heating mantle. The size of the PbSe QDs was controlled by varying the amount of injected TOP and the growth time. The QDs were purified twice by precipitating with a hexane/ethanol mixture solvent and redispersing in hexane.

### Doping of preformed PbSe QDs with manganese

We applied a diffusion doping procedure to incorporate manganese into the preformed PbSe QDs^43,44^. Mn(ac)_2_·4H_2_O (245 mg), ODE (1.5 ml), OAm (1.25 ml) and OAc (0.6 ml) were loaded into a three-neck flask and degassed at 120 °C under vacuum for 1 h. The purified PbSe QDs in hexane were added into the flask at 60 °C, and then hexane was removed completely under vacuum. The Mn incorporation was performed at 140 °C under N_2_ by injecting 0.1 M TOP-Se (79 mg Se power and 100 ml TOP) dropwise for 1 min. The Mn content in the QDs (up to 7% of all cations) was controlled by the amount of added TOP-Se (up to 0.05 mmol). The reaction was quenched by cooling to room temperature with a water bath. The QDs were purified twice by precipitating with ethanol.

### CdSe shell growth

PbSe/CdSe core/shell QDs were prepared via a cation exchange reaction^23,45^ during which the original cations in the peripheral area of the QD were replaced with Cd. For undoped QDs, CdO (160.5 mg), ODE (2 ml) and OAc (1.5 ml) were loaded into a three-neck flask, degassed at 120 °C for 15 min and then heated to 250 °C under N_2_. The mixture was maintained at this temperature until it turned into a colourless solution. The solution was then dried under vacuum at 120 °C for 1 h. The purified PbSe QDs (28.6 mg) in TCE were added in the flask at 60 °C, and then TCE was removed under vacuum. The cation exchange was performed by heating this solution at 120 °C under N_2_. The total reaction time required to achieve a 1.6-nm-thick shell was 5 h. The reaction was quenched by removing the heating mantle. The QDs were purified twice by precipitating with hexane and ethanol. For Mn-doped QDs, Mn(ac)_2_·4H_2_O (183.8 mg) and CdO (96.3 mg) were used instead of CdO (160.5 mg). The rest of the reaction was the same as for undoped QDs.

### Sample characterization

#### Optical absorption spectroscopy

Steady-state optical absorption spectra were collected using a Perkin-Elmer Lambda 950 ultraviolet–visible–NIR spectrophotometer. QDs were prepared as a dilute TCE solution and loaded into a 1-mm-thick quartz cuvette.

#### Steady-state PL spectra

NIR PL spectra were measured using a home-built PL apparatus comprising a liquid-nitrogen-cooled InSb detector, a grating monochromator and a 531 nm diode laser for QD excitation. The laser beam was mechanically chopped, and the signal was detected using a lock-in amplifier coupled to the detector. Visible PL spectra were recorded with a Horiba Scientific FluoroMax-4 spectrofluorometer using 390 nm excitation.

#### SSPD measurements of PL dynamics

In the time-resolved NIR PL measurements, QD samples were excited using either the fundamental (*hv*_p_ = 1.55 eV) or second harmonic (*hv*_p_ = 3.1 eV) output of a regeneratively amplified Ti:sapphire laser (Coherent, Mira oscillator and RegA amplifier) operating at 250 kHz (pulse width, 50 fs). The PL signal was spectrally resolved using a 20-nm-bandwidth monochromator and detected with a single-nanowire SSPD (Quantum Opus, Opus One) operating at 2.5 K. A trigger signal was provided by a photodiode, which sampled the amplifier output, and time-correlation single-photon measurements were performed using a PicoHarp (PicoQuant) photon counting module. The width of the IRF was 58 ps (full width at half maximum), which was accounted for in the data analyses^50^. PL maps (time resolved and spectral energy resolved) were obtained by recording PL dynamics for a fixed spectral energy, which was varied in 0.03 eV steps by tuning the monochromator. A series of measurements using progressively higher excitation fluences were conducted to obtain QD absorption cross-sections and biexciton lifetimes. Excitation with *hv*_p_ = 3.1 eV at very low photon densities (〈*N*_ph_〉 < 0.1) was used to generate PL traces for measuring CM efficiencies^48^. All time-resolved PL data were collected for vigorously stirred samples to avoid photocharging artefacts^48^.

#### Streak-camera measurements of NIR PL dynamics

High-resolution NIR PL dynamics were measured at the Center for Nanoscale Materials of Argonne National Laboratory. The fundamental output (800 nm, 35 fs pulses at 2 kHz) of a regeneratively amplified Ti:sapphire laser (Spectra Physics Mai-Tai oscillator, Spectra Physics Spitfire amplifier) was doubled to obtain 400 nm, 30 fs pulses. The 400 nm pump beam was loosely focused down to a ~700 μm spot onto a QD sample contained in a quartz cuvette. The sample was continuously agitated using a magnetic stirrer. The PL signal was spectrally dispersed in a Czerny–Turner spectrograph and time resolved using a NIR-sensitive streak camera equipped with a blanking unit (Hamamatsu C5680) synchronized with the Ti:sapphire oscillator. The streak camera was set to its highest resolution to measure the first 120 ps of the PL decay. The IRF was measured by recording the scattered 400 nm laser pulses. The IRF was fitted using a Gaussian profile whose full-width at half-maximum was 8 ps.

#### TA measurements

TA measurements of undoped and Mn-doped core/shell PbSe/CdSe QDs were performed using a pump–probe set-up based on a regeneratively amplified ytterbium–gadolinium tungstate (Yb:KGW) femtosecond laser (Pharos, Light Conversion) that generated ~190 fs pulses at 1.204 eV with a 2 kHz repetition rate. A fraction of the laser fundamental output (approximately half) was used to seed a high-harmonic generator (HIRO HHG, Light Conversion), producing the second harmonic radiation at ~2.41 eV, used as a pump. The remaining half of the fundamental laser output was used to seed an optical parametric amplifier (Orpheus, Light Conversion), producing the infrared 0.84 eV probe pulses. The pump beam was modulated at 1 kHz using an optical chopper. The 0.84 eV beam was split between the reference and probe channels. The pump and probe pulses were focused onto the sample so that a smaller spot illuminated by the probe beam (70 µm diameter) was centred, with the larger spot excited by the pump beam (150 µm diameter). Pump–probe delay was tuned using a 4 ns optical delay line. A matching pair of InGaAs detectors (Thorlabs) were used to measure the intensities of the reference beam and the probe beam transmitted through the sample. The detector outputs were fed into differential inputs of a lock-in amplifier (SRS SR830) synchronized with the chopper in the pump beam. TA measurements of undoped and Mn-doped CdSe QDs were performed with a white-light supercontinuum as a probe using the same set-up and the same procedures as those previously described in ref. ^29^.

#### TEM images

TEM images were taken using a JEOL 2010 microscope. TEM samples were prepared by depositing QDs onto a carbon-coated copper grid from ~10 µl of hexane solution.

## Online content

Any methods, additional references, Nature Portfolio reporting summaries, source data, extended data, supplementary information, acknowledgements, peer review information; details of author contributions and competing interests; and statements of data and code availability are available at 10.1038/s41563-023-01598-x.

### Supplementary information

Supplementary Information Supplementary Figs. 1–9, Table 1, Section 1 and references.

### Source data

Source Data Fig. 1 Numerical data.

Source Data Fig. 2 Numerical data.

Source Data Fig. 3 Numerical data.

Source Data Fig. 4 Numerical data.

Source Data Fig. 5 Numerical data.

Source Data Extended Data Fig. 2 Numerical data.

Source Data Extended Data Fig. 3 Numerical data.

Source Data Extended Data Fig. 4 Numerical data.

Source Data Extended Data Fig. 5 Numerical data.

Source Data Extended Data Fig. 6 Numerical data.

## Figures and Tables

**Extended Data Table 1:**
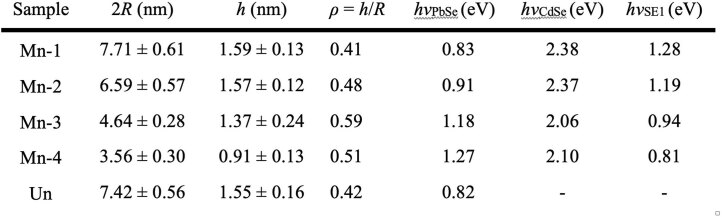
Structural and spectroscopic characteristics of the undoped and Mn-doped QDs

Size characteristics and PL energies of the Mn-doped (Mn-1 to 4) and the reference, undoped (Un) PbSe/CdSe core/shell QDs studied in the present work. Here, *R* is the overall QD radius, *h* is the CdSe-shell thickness, *ρ* is the aspect ratio defined as *ρ* = *h/R*, *hv*_PbSe_ and *hv*_CbSe_ are the spectral energies of the PbSe-core and CdSe-shell emission bands, and *hv*_SE1_ is the spectral energy of the spin-exchange PL.

## Data Availability

All data that support the findings of this study are available from the corresponding author upon request. Source data are provided with this paper.
