# Supplementary material for: Spin-exchange carrier multiplication in manganese-doped colloidal quantum dots
Source: Nat Mater. 2023 Jul 13;22(8):1013–21. doi: 10.1038/s41563-023-01598-x (PMC10390332; doi:10.1038/s41563-023-01598-x)
Supplement: Supplementary file 1 — Supplementary Figs. 1–9, Table 1, Section 1 and references. [file 41563_2023_1598_MOESM1_ESM.pdf]

nature materials

Article https://doi.org/10.1038/s41563-023-01598-x

Spin-exchange carrier multiplication in manganese-doped colloidal quantum dots

In the format provided by the authors and unedited

Table of Contents

Supplementary Section 1

Supplementary Figures 1-9

Supplementary Table 1

Supplementary References

Supplementary Section 1. Implications of Spin-Exchange Carrier Multiplication for Photovoltaics

The highest power conversion efficiency (PCE, η_{PCE}) of a photovoltaic (PV) cell in the case of fully optimized carrier multiplication (CM) is 44.4% versus 33.7% reachable without CM.[1-3] To evaluate the PCE enhancement due to CM observed in the present experiments, we introduce quantity $\delta_{PCE} = (\eta_{PCE,CM} - \eta_{PCE})/\eta_{PCE}$. In the case of 'ideal' CM, the CM onset corresponds to two bandgaps (E_g), and each increment in a photon energy of E_g above the threshold leads to a 100% increase in a quantum efficiency of photon-to-exciton conversion. In this 'ideal' situation, $\delta_{PCE} =$ 0.32 or 32%.

In principle, CM can affect the PCE via both a photocurrent and a photovoltage.[2,3] The changes in the photovoltage are expected to occur due to the increased carrier generation rate and the influence of nonradiative Auger recombination.[3] However, in the case of practically accessible multiexciton yields ($\eta_{XX} \leq 100\%$), the generated voltage is virtually unmodified by the CM process.[3] Further, the presence of CM is expected to decrease an optimal bandgap ($E_{g,opt}$) and thus the generated photovoltage compared to a no-CM case. In the case of moderate CM yields, though, the effect of CM on $E_{g,opt}$ is weak. In particular, if η_{XX} is limited by 100%, $E_{g,opt}$ is ~1 eV (see ref [2], the "M2 quantum yield model"), that is, is close to that of standard Si PVs. Importantly, as discussed later in this section, $E_{g,}$ of ~1 eV is also near a bandgap which corresponds to minimal energy losses during the SE-CM process in Mn-doped quantum dots (QDs).

Based on the above considerations, we do not expect a considerable effect of CM on the photovoltage either due to the CM-induced increase in the carrier generation rate or an excessive

photovoltage drop due to the reduced $E_{g,opt}$. Thus, the PCE enhancement occurs primarily due to the increased photocurrent. In this case, δ_{PCE} can be expressed as follows:

$$\delta_{PCE} = (\phi_s)^{-1}\Delta\phi_s = (\phi_s)^{-1}\int_0^{\infty}\eta_{XX}(\upsilon)\varphi_s(\upsilon)\,d\upsilon, \qquad (1)$$

where υ is the photon frequency, $\phi_s = \int_0^{\infty}\varphi_s(\upsilon)\,d\upsilon$ is the total incident solar flux density, $\varphi_s(\upsilon)$ is its spectral distribution, and $\Delta\phi_s$ is the flux density enhancement due to CM.

Based on the conducted measurements, the CM yield realized for the Mn-1 sample can be approximated by the dependence shown by the red line in Supplementary Fig. 8. It assumes that $\eta_{XX} = 0$ at $h\nu \leq E_{Mn,T1}$ and uses $\eta_{XX} = 0.5$ and 0.75 measured at $h\nu = 2.41$ and 3.1 eV, respectively ($E_{Mn,T1}$ is the energy of the lowest spin-flip transition of the Mn ion). Employing this functional form of $\eta_{XX}(\upsilon)$, we obtain the spectral dependence of flux density enhancement, $\Delta\varphi_s = \eta_{XX}(\upsilon)\varphi_s(\upsilon)$ (highlighted by red shading in Supplementary Fig. 8), and then evaluate δ_{PCE} from eq 1, which yields $\delta_{PCE} = 8.5\%$.

The computed value of δ_{PCE} likely underestimates the actual PCE enhancement as the utilized $\eta_{XX}(\upsilon)$ neglects the contribution from ordinary CM at energies between $2E_g$ (1.66 eV) and $E_{Mn,T1}$ (2.1 eV), and further assumes a gradual (linear) growth of η_{XX} at $h\nu > E_{Mn,T1}$. However, even this lower bound of δ_{PCE} is quite impressive, as it is approximately a quarter of the maximal theoretical enhancement attainable with CM.

Next, we consider the model situation when the multiexciton yield of spin-exchange (SE) CM is described by a step-like dependence for which η_{XX} is 1 at energies above $E_{Mn,T1}$ and 0 below it (black dashed line in Supplementary Fig. 8). To maximize the photovoltage and still allow for SE-

CM, the PbSe core bandgap should be around $E_{Mn,T1}/2$, that is, $E_g \approx 1.05$ eV. For these parameters, $\delta_{PCE} = 18\%$, which is more than a half of the maximal PCE improvement allowed by CM.

One potential complication for practical applications of Mn-doped PbSe/CdSe QDs studied in the present work is a large potential barrier for extraction of holes that are localized within the PbSe core. It is likely a lesser problem in solution-based photochemistry where holes need to traverse across a single, fairly thin CdSe layer. However, it might complicate applications in PVs that require long-range hole transport. This problem can, potentially, be overcome using, for example, 'inverted' CdSe/PdSe structures wherein holes are localized in the shell region while electrons are still delocalized across both the core and the shell.

To further optimize SE-CM for applications in solar photoconversion, one can, in principle, explore magnetic dopants other than Mn. One such possibility is Co^{2+} ions. As Mn ions, these dopants enter II-VI and IV-VI semiconductors as isovalent substitutional impurities that exhibit a comparable strength of exchange coupling.[4] However, a potential advantage of Co^{2+} dopants is that the energy of their spin-flip transition ($E_{Co} \approx 1.7$ eV; ref. [5]) is appreciably lower than that of Mn, making them better suited for applications in solar energy conversion.

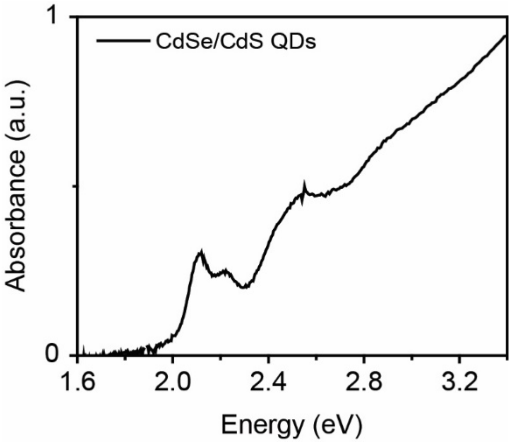

Supplementary Figure 1. The absorption spectrum of the undoped CdSe quantum dots (QDs; 2.2-nm overall radius) with a thin protective CdS shell (~1 semiconductor monolayer thickness), which have been used in the study of Auger dynamics shown in Fig. 1b of the main article.

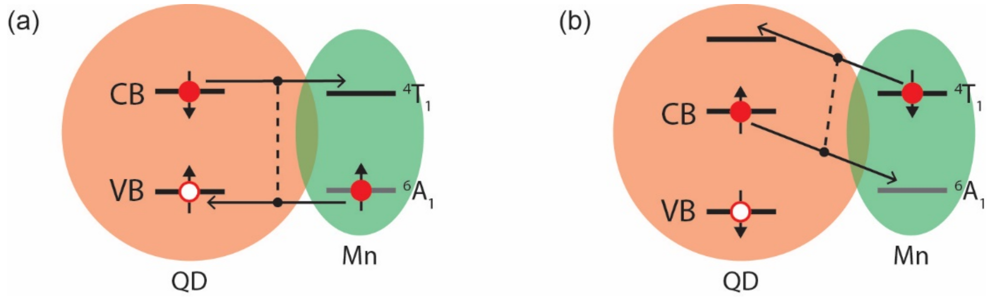

Supplementary Figure 2. (a) A spin-exchange representation of single-exciton QD-Mn transfer. This process can be described as concerted spin-conserving transfer of two electrons. During the first step, the spin-up electron from the $^6A_1$ ground state of the Mn ion is transferred to the QD to fill the valence-band (VB) hole. In the second step, the spin-down QD conduction-band (CB) electron is transferred to the Mn ion to fill the vacancy in its 3d shell, which creates the excited $^4T_1$ state. The $^6A_1$ ground state of the Mn ion comprises 5 coaligned spin-up electrons in the 3d shell. In the excited $^4T_1$ state, the spin of one of the electrons is flipped, that is, the 3d shell comprises 4 spin-up electrons and 1 spin-down electron. (b) Schematic depiction of spin-exchange Auger recombination of a hybrid biexciton comprising a QD exciton and an excited Mn ion (Mn*). It occurs via two concerted electron-transfer steps. The first step is transfer of a spin-down electron from the excited Mn $^4T_1$ state to a higher-energy ('hot') QD CB state, which leaves a vacancy in the Mn 3d shell. In the second step, this vacancy is filled via transfer of a spin-up electron from the band-edge QD CB state, which recovers the ground $^6A_1$ state of the Mn ion.

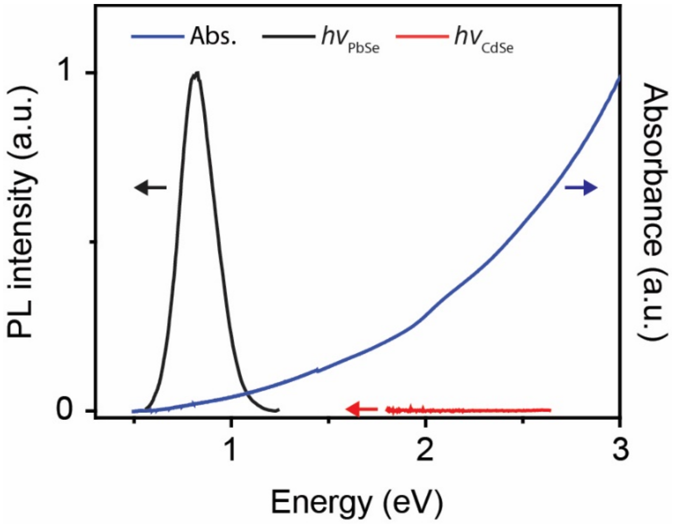

Supplementary Figure 3. Absorption (blue) and photoluminescence (PL) (red and black) spectra of undoped PbSe/CdSe core/shell QDs (reference sample).

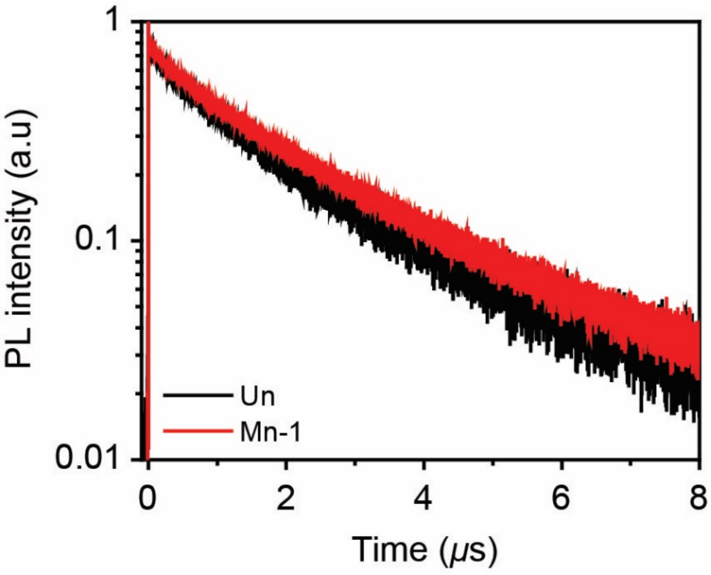

Supplementary Figure 4. PL decay of band-edge near-infrared (NIR) emission of Mn-doped (Mn-1, red) and undoped (Un, black) PbSe/CdSe core/shell QDs. The averaged PL lifetimes are 2.0 μs and 1.7 μs for the doped and the undoped QDs, respectively.

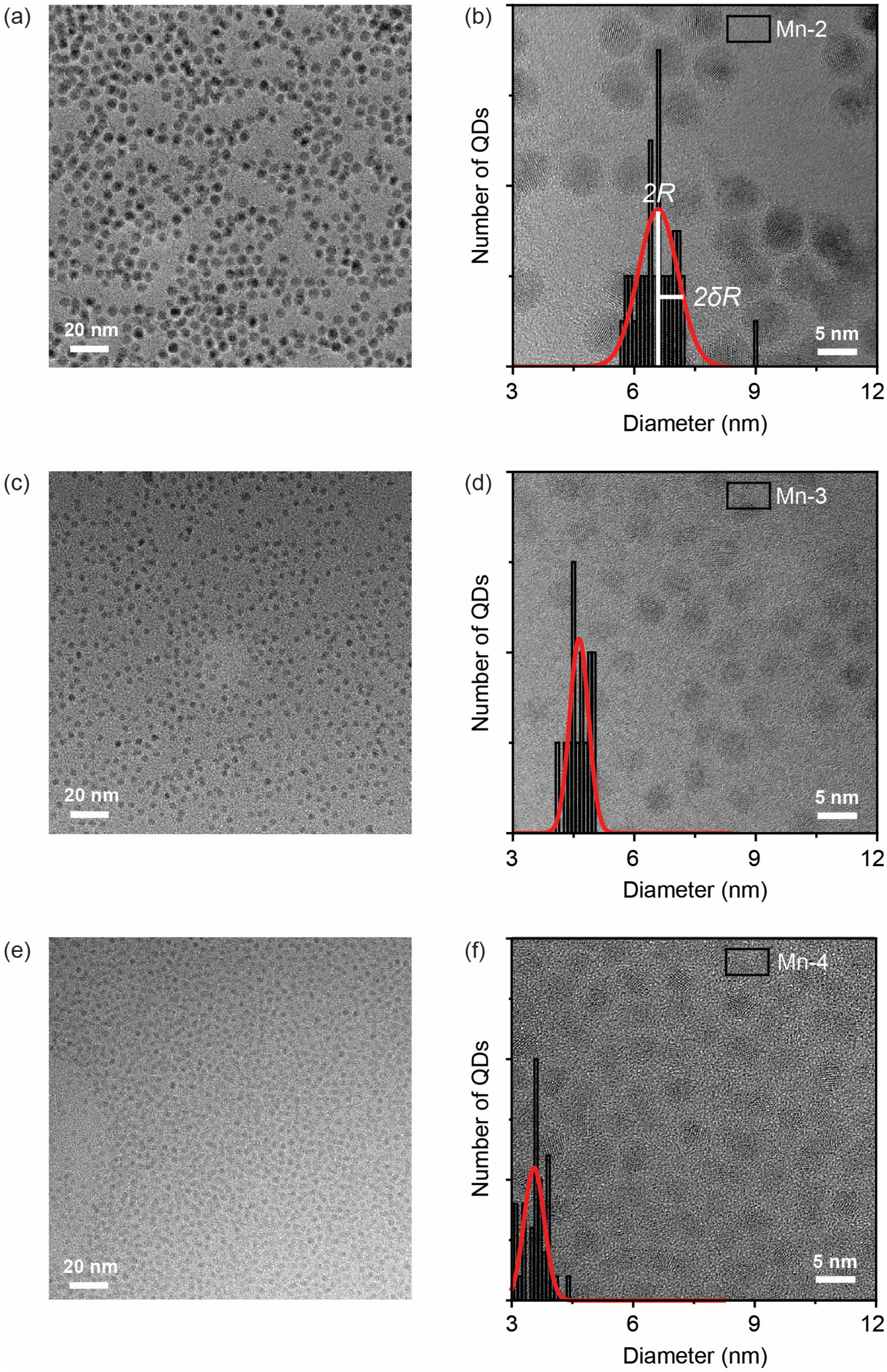

Supplementary Figure 5. Transmission electron microscopy images and size distributions of size-controlled Mn-doped PbSe/CdSe core/shell QDs. (a,b) Sample Mn-2; $2R =$ 6.59 nm and $2\delta R$ = 0.57 nm. (c,d) Sample Mn-3; $2R =$ 4.64 nm and $2\delta R$ = 0.28 nm. (e,f) Sample Mn-4; $2R$ = 3.56 nm and $2\delta R =$ 0.30 nm.

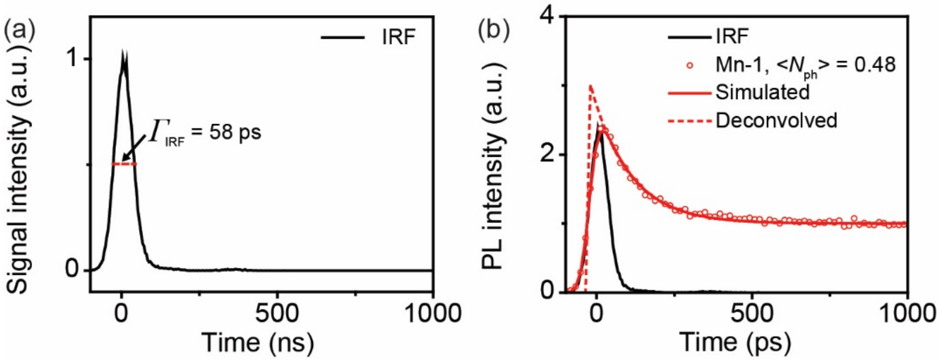

Supplementary Figure 6. (a) Instrument response function (IRF) of the time-resolved PL apparatus based on a superconducting single-photon detector (SSPD) used in the CM measurements. The IRF full width at half maximum (Γ_{IRF}) is 58 ps. (b) An example of deconvolution of a 'raw' experimental trace (red circles; <N_{ph}> = 0.48, $h\nu_p$ = 1.20 eV) using the measured IRF (black line) which yields a 'true' shape of the PL time transient (dashed red line). The trace obtained by applying the IRF to the extracted time transient (solid red line) perfectly matches the experimental trace (red circles).

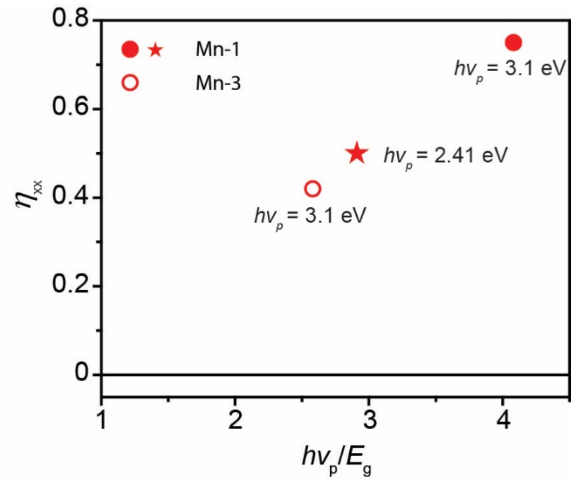

Supplementary Figure 7. It is instructive to compare CM yields realized for sample Mn-1 with 3.1 eV (red solid circle) and 2.41 eV (red star). With 3.1-eV pump photons used in the PL measurements, we can access both the $^4T_1 - {}^6A_1$ and $^4T_2 - {}^6A_1$ transitions of the Mn ion (Fig. 4b). The energies of both of these transitions are sufficient to drive SE-CM in sample Mn-1 as $E_{Mn,T1,2} > 2E_g$ (= 1.66 eV). However, the pump photons used in the TA studies ($h\nu_p$ = 2.41 eV) can excite only the $E_{Mn,T1}$ transition, which should reduce the CM efficiency due to elimination of the $E_{Mn,T2}$ CM pathway. In agreement with this expectation, the CM yield measured with the 3.1 eV photons (η_{XX} = 75%; red solid circle) is 1.5 times higher than that realized with $h\nu_p$ = 2.41 eV (η_{XX} = 50%; red star). Interestingly, η_{XX} observed for sample Mn-1 with 2.41-eV excitation is comparable to that measured for sample Mn-3 with 3.1 eV photons (red open circle). Despite the difference in pump photon energies, in both cases, SE-CM is driven by a single Mn* transition ($^4T_1 - {}^6A_1$ for sample Mn-1 and $^4T_2 - {}^6A_1$ for sample Mn-3), which leads to similar CM yields.

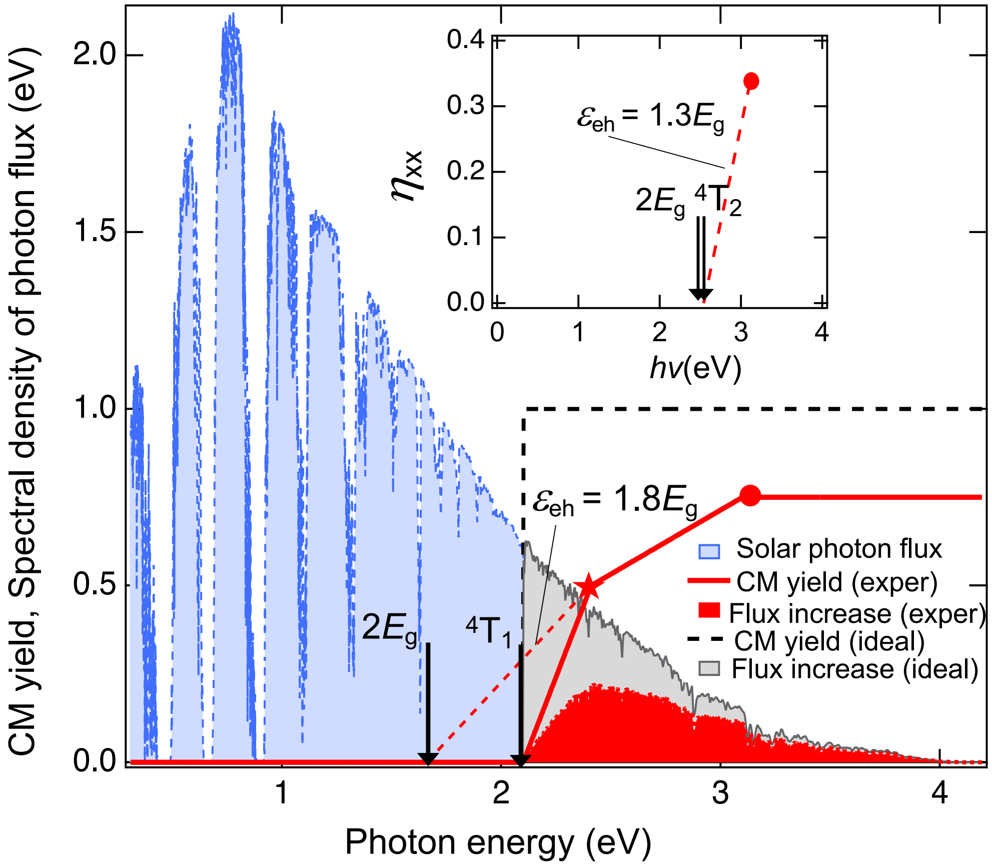

Supplementary Figure 8. Experimentally measured CM yields of sample Mn-1 (red symbols) are approximated using a linear interpolation (solid red line). The blue spectrum is the spectral distribution of the sunlight photon flux. The spectrum shown by red shading is the effective solar flux enhancement obtained using experimentally measured CM yields. The 'ideal' SE-CM yield is shown by the dashed black line. It assumes, that the SE-CM onset occurs at $h\nu = E_{\mathrm{Mm,T1}}$, following which $\eta_{xx} = 1$. The corresponding flux enhancement is shown by gray shading. The dashed red line is used to estimate the upper limit of ε_{eh}. Inset: The red circle is the measured CM yield for sample Mn-4. The dashed line is used to estimate ε_{eh}.

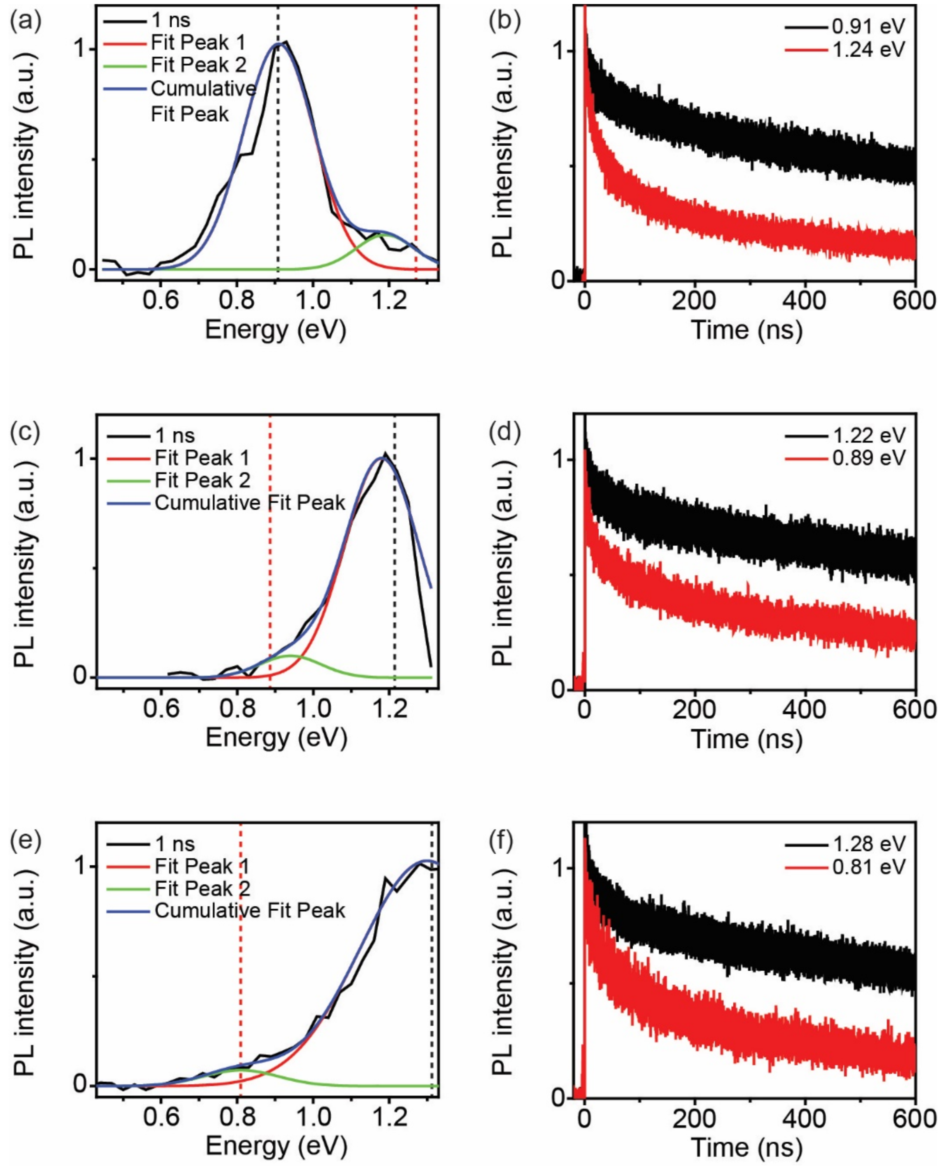

Supplementary Figure 9. PL spectra of samples (a) Mn-2, (c) Mn-3, and (e) Mn-4 measured at time delay of 1 ns after excitation with a 3.1 eV laser pulse (black). These spectra are fitted to a sum of two Gaussian bands that correspond to the core ($h\nu_{PbSe}$, red) and SE ($h\nu_{SE1}$, green) PL bands. PL dynamics for samples (b) Mn-2, (d) Mn-3, and (f) Mn-4 for the core and SE bands (the detection energies are shown by the vertical black and red dashed lines, respectively).

Supplementary Table 1. Biexciton yields (η_{XX}) of the Mn-doped and undoped (reference) PbSe/CdSe core/shell QDs observed for pump photon energies of 3.1 eV and 2.41 eV.

Sample	E_g (eV)	η_{XX} (3.1 eV)	η_{XX} (2.41 eV)
Mn-1	0.83	0.75	0.50
Mn-3	1.18	0.42	-
Mn-4	1.27	0.33	-
Un	0.82	0.48	0.23

Supplementary References

1. Shockley, W., Queisser, H. J. Detailed balance limit of efficiency of p-n junction solar cells. *J. Appl. Phys.* **32**, 510-519 (1961).

2. Hanna, M. C., Nozik, A. Solar conversion efficiency of photovoltaic and photoelectrolysis cells with carrier multiplication absorbers, *J. Appl. Phys.* **100**, 1-8 (2006).

3. Klimov, V. I. Detailed-balance power conversion limits of nanocrystal-quantum-dot solar cells in the presence of carrier multiplication. *Appl. Phys. Lett.* **89**, 123118 (2006).

4. Nawrocki, M., Hamdani, F., Lascaray, J. P., Golacki, Z., Deportes, J. Ion-carrier electron exchange constants for CdCoSe semimagnetic semiconductor. *Solid State Commun.* **77**, 111-114 (1991).

5. Yang, J. et al. Co^{2+}-doping of magic-sized CdSe clusters: Structural insights via ligand field transitions. *Nano Lett.* **18**, 7350–7357 (2018).
